# The Chick Chorioallantoic Membrane Model: A New In Vivo Tool to Evaluate Breast Cancer Stem Cell Activity

**DOI:** 10.3390/ijms22010334

**Published:** 2020-12-30

**Authors:** Marta Teixeira Pinto, Ana Sofia Ribeiro, Inês Conde, Rita Carvalho, Joana Paredes

**Affiliations:** 1 i3S—Institute of Investigation and Innovation in Health, 4200-135 Porto, Portugal; mtpinto@i3s.up.pt (M.T.P.); aribeiro@ipatimup.pt (A.S.R.); iconde@ipatimup.pt (I.C.); rcarvalho@ipatimup.pt (R.C.); 2 Ipatimup, Institute of Molecular Pathology and Immunology, University of Porto, 4200-135 Porto, Portugal; 3 Faculty of Medicine, University of Porto, 4200-319 Porto, Portugal

**Keywords:** cancer stem cells, chicken chorioallantoic membrane, in vivo model, limiting dilution assay

## Abstract

The high plasticity of cancer stem-like cells (CSCs) allows them to differentiate and proliferate, specifically when xenotransplanted subcutaneously into immunocompromised mice. CSCs are highly tumorigenic, even when inoculated in small numbers. Thus, in vivo limiting dilution assays (LDA) in mice are the current gold standard method to evaluate CSC enrichment and activity. The chick embryo chorioallantoic membrane (CAM) is a low cost, naturally immune-incompetent and reproducible model widely used to evaluate the spontaneous growth of human tumor cells. Here, we established a CAM-LDA assay able to rapidly reproduce tumor specificities—in particular, the ability of the small population of CSCs to form tumors. We used a panel of organotropic metastatic breast cancer cells, which show an enrichment in a stem cell gene signature, enhanced CD44^+^/CD24^−/low^ cell surface expression and increased mammosphere-forming efficiency (MFE). The size of CAM-xenografted tumors correlate with the number of inoculated cancer cells, following mice xenograft growth pattern. CAM and mice tumors are histologically comparable, displaying both breast CSC markers CD44 and CD49f. Therefore, we propose a new tool for studying CSC prevalence and function—the chick CAM-LDA—a model with easy handling, accessibility, rapid growth and the absence of ethical and regulatory constraints.

## 1. Introduction

Cancer stem-like cells (CSCs), also designated as tumor-initiating cells (TICs), were shown to be critical in inducing tumor heterogeneity, but also in inducing cancer development, progression and metastasis due to their capacity to be chemoresistant, invasive and dormant [1,2]. These cells possess the ability to self-renew, harboring high levels of plasticity and being able to differentiate in different phenotypic populations, especially when engrafted in in vivo models, such as immunocompromised mice. It has been also shown that CSC populations are also enriched in human metastatic lesions [3,4], as well as in metastatic cancer cell lines [5,6,7], being responsible for therapy resistance.

CSCs have been described as representing a small population of the tumor tissue and expressing specific cell surface markers. In breast cancer, it was shown that cancer cells expressing CD44^+^/CD24^−/low^ are particularly efficient in forming new tumors, showing the capacity to self-renew and to be therapy-resistant [8]. However, in the last years, several other surface proteins have been indicated as putative biomarkers for breast CSCs, such as CD49f [9] and EpCAM [10], which are usually used in combination to characterize mammary stem cell subpopulations [11]. Breast cancer cells with high ALDH1 activity also demonstrate stem cell properties, namely by contributing to metastases and chemotherapy resistance [12,13], as well as the ones co-expressing P-cadherin, CD44 and CD49f, especially in basal-like breast cancer [14,15]. Therefore, these markers are widely used to perform the in vitro characterization of breast CSCs [1,15,16,17], which can be combined with their mammosphere-forming efficiency. Actually, the ability of cells to form multicellular spheroids is the classic in vitro assay to evaluate stem cell properties, which takes advantage of the fact that only cells with stem-like properties are able to survive and proliferate in the absence of attachment to an exogenous substratum [18]. As such, “mammospheres” are highly enriched in mammary stem cells or breast CSCs. Moreover, single cells isolated from mammospheres can generate multilineage colonies when cultured in the presence of serum on a collagen substratum that promotes their differentiation. Mammosphere-derived cells are also capable of generating clonally complex functional structures in reconstituted 3D culture systems, such as Matrigel [1,16,17,18].

Importantly, one of the most significant drivers of the malignant phenotype is the tumor microenvironment. The components of the extracellular matrix (ECM) and other microenvironment constituents (cellular elements and soluble factors) can also modulate cancer stemness. Importantly, it affects the oxygen concentration, pH and nutrient availability, which will, in turn, modulate the CSC activity and functions (such as self-renewal and chemoresistance) [19], as well as influence the breast cancer stemness and tumor aggressiveness [1]. In order to provide the microenvironmental cues, it is therefore critical to evaluate CSCs in an in vivo context. To assess the tumorigenic capacity, breast cancer cells are traditionally xenotransplanted into the subcutaneous region under the mammary fat pad of female immunocompromised mice in varying cell dilutions [6,15,20,21,22]. Although these encompass ethical and financial limitations, the limiting dilution assays in mice are the “gold standard” method to evaluate CSC tumorigenicity.

In contrast, the chick embryo chorioallantoic membrane (CAM) is a low cost, reproducible and reliable model used to investigate several functional features of tumor biology, such as the ability of cells to be tumorigenic, to invade and to metastasize into the embryo [23,24]. The CAM is a highly vascularized extraembryonic membrane, thus providing rich nutrient conditions for the spontaneous growth of human tumor cells. Actually, this model has been used for many years to support the growth of numerous types of tumors, including breast cancer [17,25,26,27,28,29]. Additionally, supporting tumor growth, the CAM has a deficient immunological and inflammatory response in earlier stages of the chick development [27,30]. Moreover, it has also been used as a platform to analyze the values of anticancer drugs [29,31] and radiation therapy [30].

Taking into account the several described advantages of the CAM model, our major goal was to develop a new limiting dilution assay in vivo model able to rapidly reproduce tumor specificities—in particular, the ability of the small population of CSCs to form tumors in an immuno-incompetent host. Thus, in this manuscript, we describe, for the first time, the use of a limiting dilution assay to evaluate cancer cells’ stemness in the chick CAM, in comparison with the in vivo gold standard mice model, using a panel of organotropic MDA-MB-231 metastatic breast cancer cells. We demonstrated that the size of the tumors formed in the CAM correlates with the number of inoculated cells, following the same cell growth pattern as in mice. Furthermore, the CAM and mice tumors were histologically comparable and displayed a similar pattern of breast CSC markers. These results allowed us to propose that the chick CAM model can be a new tool to evaluate breast CSC properties in vivo.

## 2. Results

### 2.1. Organotropic Breast Cancer Cells Show Increased In Vitro CSC Properties

**Figure 1:**
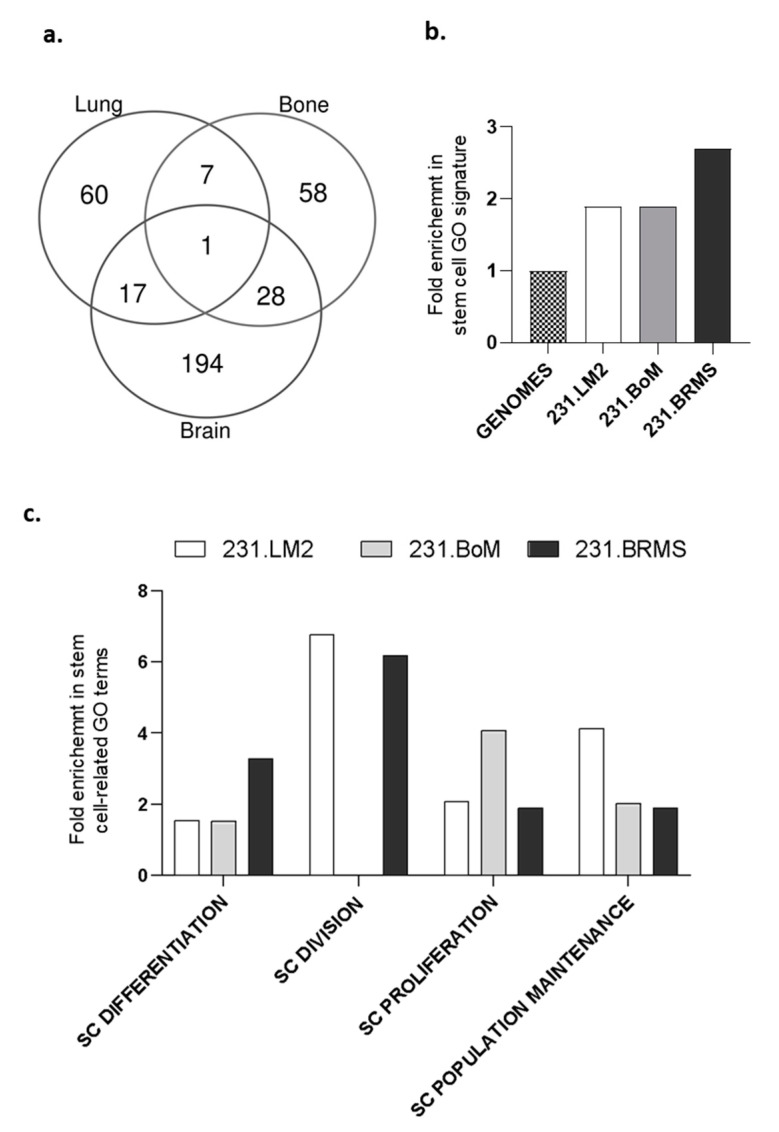
Organotropic breast cancer cell lines show an enriched stem cell-like signature. Enrichment analysis for stem cell gene ontology terms using the significantly deregulated gene (DEG) list between each organotropic breast cancer cell line and the parental MDA-MB-231 cell line. (**a**) Venn diagram showing the common and specific DEGs between the different organotropic variants. (**b**) Overall enrichment in stem cells gene ontology (GO) signature and (**c**) in stem cell-related GO terms after an analysis of the specific DEGs annotated with a stem cell function. SC: stem cell.

Human metastatic lesions have been described as being enriched in cancer cells with stem cell properties. Therefore, we used the highly metastatic human breast cancer cell line MDA-MB-231 and its organotropic variants (lung, bone and brain) [32,33,34]. As a first approach to validate their stem cell properties, we performed an in-silico analysis to evaluate the prevalence of a stem cell signature in each of the breast cancer cell lines. We crossed the list of genes previously described as significantly deregulated in each one of the organotropic-derived breast cancer cell lines when compared with the parental 231 breast cancer cell line [32,33,34] (resumed in Supplementary Table S1), in order to obtain both the specific, and the shared, list of deregulated genes. From this analysis, 80.8% (194 out of 240) of the genes were specifically deregulated in brain metastatic breast cancer cells, compared with 70.5% (60 out of 85) and 62.1% (59 out of 95) specifically deregulated genes for bone and lung metastatic cells, respectively (Figure 1a).

Further, we determined the stem cell enrichment for each organotropic breast cancer cell line using the specifically deregulated gene lists by analyzing the stem cell gene ontology (GO) terms retrieved from http://amigo.geneontology.org/amigo/search/bioentity?q=stem. As expected, our results showed that all organotropic breast cancer cells were enriched in genes associated with stem cell functions (Figure 1b)—particularly, the 231.BRMS cell line. Concerning the different stem cell GO terms—SC population maintenance, SC proliferation, SC division and SC differentiation—major differences were observed within the different organotropic breast cancer cells (Figure 1c). 231.LM2 is particularly enriched in SC division and population maintenance, 231.BoM is mainly enriched in SC proliferation and 231.BRMS is enriched in SC division and differentiation (Figure 1c).

**Figure 2:**
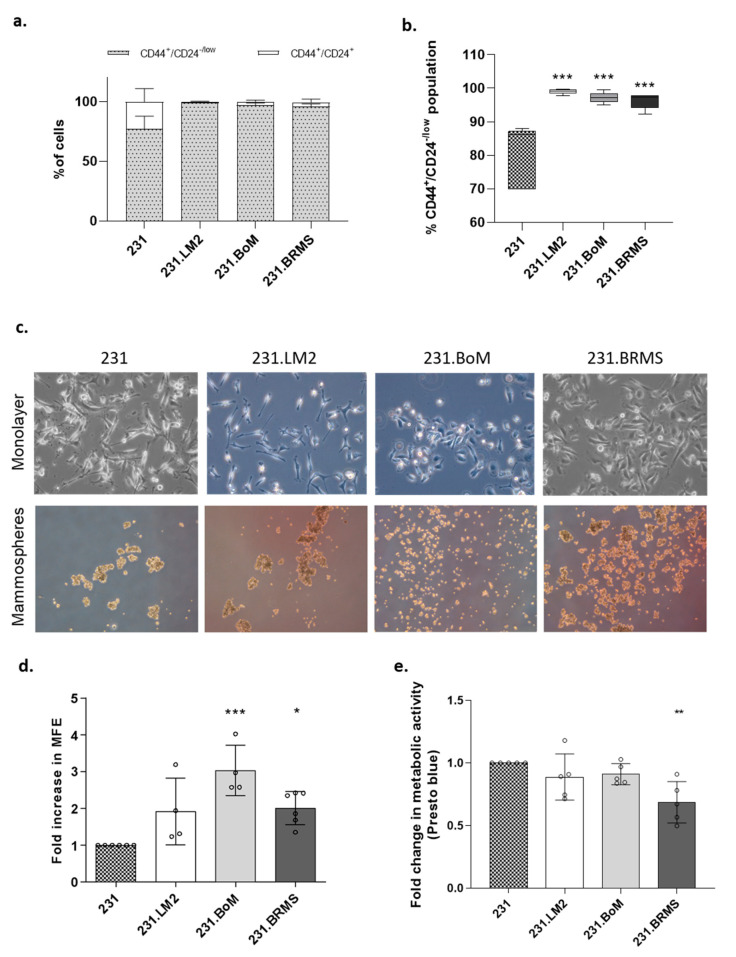
Organotropic breast cancer cells show increased in vitro cancer stem cell (CSC) properties. (**a**) Characterization of the cancer cell populations for CD44 and CD24 CSC markers by flow cytometry analysis in 231 breast cancer cells and its organotropic variants. (**b**) Percentage of the CSC population CD44^+^/CD24^−/low^ in 231 breast cancer cells and its organotropic variants. (**c**) Representative picture of monolayer (2D) and mammospheres (20× magnification). (**d**) Mammosphere-forming efficiency (MFE) in organotropic breast cancer cells (minimum of 4 biological replicates) compared with parental cells. (**e**) Fold change in cell viability and metabolic activity measured by the Presto blue assay. Five biological replicates; *—*p* < 0.05, **—*p* < 0.01 and ***—*p* < 0.001.

Finally, to validate in vitro the enriched cancer stem cell (CSC) phenotype in the different organotropic metastatic breast cancer cell lines, we performed the in vitro gold-standard methods for CSC identification: characterization in terms of breast CSC markers and the mammospheres-forming assay, which measures the ability of cells to be anoikis-resistant and to grow in anchorage-independent conditions, as well as their capacity to self-renew and differentiate. In particular, the CD44^+^CD24^−^ phenotype was measured by flow cytometry. Although all the cell lines were constituted by a large subpopulation of CD44^+^/CD24^−/low^ cells, it was still possible to observe a very significant enrichment in the CD44^+^/CD24^−/low^ subpopulation in the organotropic variants when compared with the parental cell line (with 80.1% in 231, 99% in 231.LM2, 97.2% in 231.BoM and 96.3% in 231.BRMS) (Figure 2a,b). The enriched stem-like phenotype in the organotropic breast cancer cell lines was further confirmed by the observed increased mammosphere-forming efficiency (MFE, although this effect was only significant in the 231.BoM and 231.BRMS cells when compared with the 231 parental cell line) (Figure 2c,d). Of notice is that these changes were not due to differences in the proliferative capacity of the cells (Figure 2e).

### 2.2. Establishing a Limiting Dilution Assay for CSC Identification Using the In Vivo Chicken Egg CAM Model

**Figure 3:**
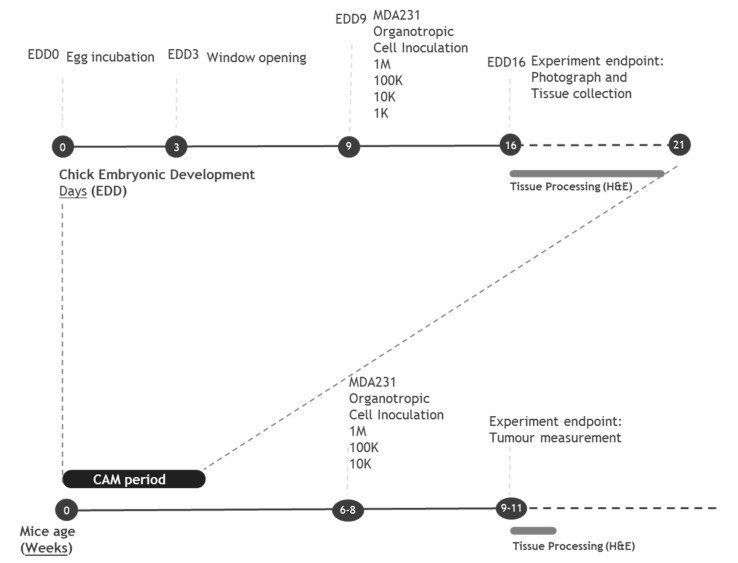
In vivo limiting dilution assay (LDA) workflow: a timeline of the chorioallantoic membrane (CAM) and mice experiments. Fertilized eggs are incubated for 3 days; at which time, a window in the shell is opened. At embryonic development day 9 (EDD9), breast cancer cells are inoculated on top of the CAM. At EDD16, eggs are sacrificed, and the tumor growth is examined. Mice with 6–8 weeks of age are subcutaneously injected with breast cancer cells. Tumor growth is monitored for 3 weeks. At weeks 9–11, mice are sacrificed and tumors are further examined.

Our main aim was to test the ability of organotropic breast metastatic cells to grow in the non-mammalian chick embryo chorioallantoic membrane (CAM). For that, we adapted the concept of the in vivo limiting dilution assay, which is normally used to determine the cancer-initiating cell frequency of an established suspension cell line. For a proper comparison, we used both CAM and immunocompromised mice xenograft models. Breast cancer cell lines were inoculated at different concentrations in the CAM and in mice, according to the experimental design depicted in Figure 3.

**Figure 4:**
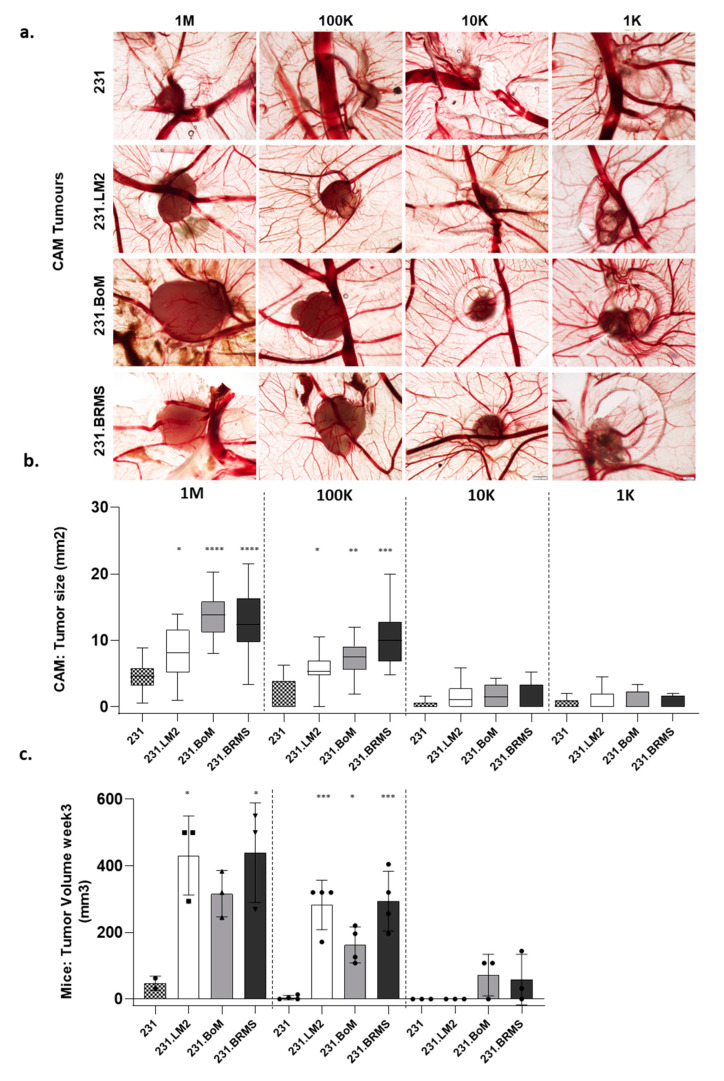
Organotropic breast cancer cells display an increased tumorigenic ability in the choriallantoic membrane of the chick embryo (CAM). (**a**) Representative pictures of CAM xenografted tumors (4 cell lines × 4 cell dilutions); scale bar = 500 ¼m. (**b**) Tumor size evaluation in the LDA performed in the CAM by inoculations of 1-M, 100-K, 10-K and 1-K cells per egg. (**c**) Tumor size evaluation in a LDA performed in immunodeficient mice by subcutaneous injection of 1-M, 100-K and 10-K cells number per animal. *—*p* < 0.05, **—*p* < 0.01, ***—*p* < 0.001 and ****—*p* < 0.0001).

Although all cell lines were able to form tumors in the CAM, a different phenotype was observed between the parental 231 and the organotropic variants. The parental cell line (231) typically formed disaggregated and diffuse tumors, whereas the organotropic variants formed significantly bigger and cohesive tumors (Figure 4a). Further, as described in the previous section, we performed a limiting dilution assay (LDA), in both the CAM and mice, to evaluate the stem cell frequency of the organotropic breast cancer cells. We evaluated the tumor size (Figure 4b,c) and the frequency of tumor formation (Table 1 and Table 2). For the CAM-LDA, 18 embryos were inoculated with 1-M cells, 9–11 embryos with 100 K, 10 to 11 with 10-K cells and 8–10 embryos with 1-K cells. All organotropic cells showed significantly bigger tumors when inoculated at 1-M and 100-K cells in the CAM (Figure 4b) in compassion to the parental cell lines. Both the in vitro and in vivo CAM results were consistent with each other. The next step was to validate them using a limiting dilution assay in the in vivo mice model, since this is the gold standard method in the CSC field to prove the stem-like ability of cancer cells. For mice LDA, two–four mice were used per condition (dilution/cell line), and, after three weeks, organotropic cells also showed significantly bigger tumors then the parental cell line 231 when inoculated at 1-M (231.LM2 and 231BRMS) and 100-K (all three cell lines). As in the CAM, no differences were detected when 10-K cells were inoculated; to reduce the number of used animals, the 1-K dilution was not tested in mice (Figure 4c). Though the tumor size differences were not maintained at the lower cell inoculations (10-K and 1-K cells), it was still possible to observed an increase in the frequency of tumor formation (Table 1) that reflected a significant increase in the stem cell frequency of all organotropic breast cancer cells, as calculated by Extreme Limiting Dilution Analysis (ELDA) software [35]. Interestingly, and in accordance with the mammosphere-forming assay, this effect showed to be more pronounced in 231.BoM and 231.BRMS. The number of tumor-initiating cells was higher in 231.BoM and 231.BRMS, as already shown in the previously described CAM-LDA assay. Although with less significance, the same analysis performed for mice LDA showed the same pattern for stem cell frequency (Table 2). In summary, the in vivo chick CAM model recapitulates the in vitro CSC properties of breast cancer cells, producing tumors with sizes and stem cell frequencies comparable with the ones found with mice LDA assay.

### 2.3. Validation of Breast CSC Markers in CAM and Mice Xenografted Tumors

**Figure 5:**
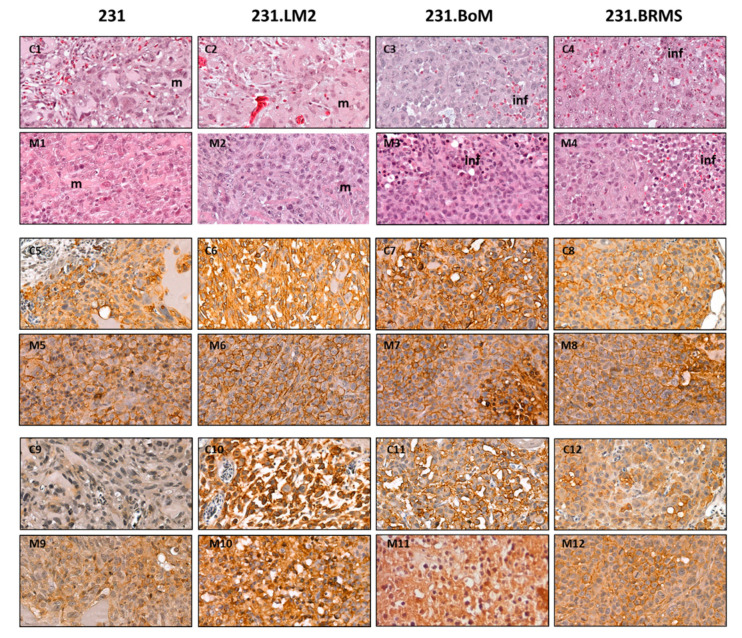
CAM and mice xenografts display similar citocellular organization and stem marker patterns. Each column regards a cell line (231, 231.LM2, 231.BoM and 231.BRMS). (**C1**–**C4**) Hematoxylin–eosin staining in the CAM xenografted tumors (63× magnification); m—Matrigel and inf—infiltrate. (**M1**–**M4**) Hematoxylin–eosin staining in the mice xenografted tumors (63× magnification). (**C5**–**C8**) CD44 labeling in CAM tumors (63 magnification). (**M5**–**M8**) CD44 labeling in mice tumors (63× magnification). (**C9**–**C12**) CD49f labeling in CAM tumors (63× magnification). (**M9**–**M12**) CD49f labeling in mice tumors (63× magnification).

In addition to the previously described macro analysis, the tumors derived from 1-M cells were recovered in both in vivo models and processed for histological analysis. Immunohistochemistry labeling was performed for the CD44 and CD49f breast CSC markers (Figure 5).

In the CAM, tumors derived from all cell lines were able to invade the CAM and to be vascularized (see Supplementary Figure S1 at 10× magnification). Cellular morphology in the CAM (Figure 5C1–C4) and mice (Figure 5M1–M4) tumors could be compared. The tumors derived from the parental cell line (231) present a clear Matrigel component (m in Figure 5C1,M1), in contrast with the organotropic cell lines, which show a more compact and homogeneous aspect without the detectable presence of Matrigel (Figure 5C2–C4,M2–M4). In agreement with the in vitro results, organotropic breast cancer cell lines show intense and strong staining for both CD44 (Figure 5C6–C8 in comparison to C5 and Figure 5M6–M8 in comparison to M5) and CD49f (Figure 5C10–C12 in comparison to C9 and Figure 5M10–M12 in comparison to M9).

## 3. Discussion

Tumor progression is a complex multistep process and cancer cells have to display great plasticity in order to survive to the metastatic stepwise cascade [36]. In fact, primary tumors are known to be biologically heterogeneous and composed of different subpopulations of cancer cells with distinct properties, allowing them to adapt to the different processes of the tumor progression cascade. Contributing to this process, the presence of a small subpopulation of cancer cells within the tumor bulk with stem-like properties was demonstrated—cancer stem cells (CSCs) [37]. CSCs are crucial for disease progression, given their self-renewal, plasticity and differentiation capabilities. They are able to induce tumorigenesis, forming or repopulating heterogeneous cancer cell populations [3,14,15,38]. Besides their highly tumorigenic potential, CSCs are resistant to most treatments currently available, and this drug-resistance can promote metastasis and tumor relapses. Therefore, CSC research is an urgent topic in oncology and new and faster tools to evaluate the stem profiles of tumor cells are crucial.

Currently, a combination of in vitro and in vivo assays are used as standard methods to evaluate the self-renewal and differentiation potential of CSCs. Specifically, in breast cancer, an enrichment in CSCs can be identified in vitro by the CD44^+^/CD24^−/low^ profile and by the formation of mammospheres [18] and by in vivo limiting dilution assays in mice models. Although these mice xenograft models are considered the “gold standard” method to evaluate stemness in vivo, they are invariably associated with high production costs and strict ethical considerations.

Here, we aimed to validate a new in vivo LDA based on the chick embryo chorioallantoic membrane (CAM) as an alternative research tool to be used in the CSC research field. In particular, we demonstrated the ability of the small population of CSCs to form tumors in the CAM, an immuno-incompetent host. In contrast to the mice model, the CAM model has low costs and little regulatory issues; it is also a reproducible and reliable tool to study several aspects of tumor biology, since it has been widely used to study tumor growth and behavior, therapy delivery and efficiency, angiogenesis and metastasis [3,17,23,24,25,26,27,28].

To establish the new LDA in the chick CAM, a panel of organotropic breast cancer cells was used as cell models. In order to determine if the CAM model would respond to the constraints faced in the in vivo model, we started by characterizing the CSC phenotype of breast cancer cells, previously established by Massagué [32,33,34], that show tropism to different organs (lung, bone and brain). All organotropic breast cancer cells were shown to be enriched in genes associated with stem cell features. In addition, all of them showed increased in vitro CSC activity when compared with the parental cell line, measured either by the CD44^+^/CD24^−/low^ profile and by the increased mammosphere-forming efficiency. This effect was more prominent with the bone (231.BoM) and brain (231.BRMS) organotropic breast cancer cells.

Following the in vitro characterization for stem cell features, in vivo LDA was performed in the CAM and in mice. Note that the chick embryo is naturally immune-incompetent, thus allowing the grafting of human tumor cells. Our results in the CAM showed that all cell lines were able to form tumors in seven days, but the organotropic variants formed significantly larger and more compact tumors than those from the parental cell line. In agreement with the previously established stem cell profile, 231.BoM and 231.BRMS formed more prominent tumors. Using mice LDA, tumor formation took three weeks, and the organotropic cell lines were also clearly more tumorigenic; however, for the 1-M cells, differences could only be detected for the 231.LM2 and 231. BRMS cell lines. By using the CAM model, it was possible to achieve 9–18 samples per condition (in contrast with 2–4 in the mice model), thus rendering the statistical analysis more robust in the CAM than in mice LDA. This is clear by the significance levels obtained (that were higher in the CAM-LDA than in mice LDA) but, also, by the consistency obtained between dilutions. Note that 100-K derived tumors were smaller than the 1-M cell derived tumors but followed the same growth pattern between the cell lines.

Besides the tumor size, the stem cell frequency of the organotropic breast cancer cells was determined based on the frequency of tumor formation for each dilution in CAM and mice assays [35]. Consistently with in vitro characterization, all organotropic breast cell lines presented significantly higher stem cell frequency in comparison with the parental cell line when these were inoculated in the CAM. However, for mice LDA, significance was only achieved for 231.BoM and 231.BRMS. Still, the magnitude of the values obtained in the CAM were comparable to the ones found in mice LDA. This analysis demonstrates the importance of a robust set of samples, not always possible to achieve in mice assays. In summary, the in vivo chick CAM model recapitulates the in vitro CSC properties of breast cancer cells, producing tumors with sizes and stem cell frequencies comparable with the ones obtained with the in vivo mice LDA assay.

Finally, xenografted CAM and mice tumors were further analyzed in terms of cell morphology (Hematoxylin–eosin (H&E) staining) and expression of breast CSC markers CD44 and CD49f. As in mice, the morphology of CAM xenografted tumors resembles cancer patient tumors by containing extracellular matrix, stromal cells and extensive vasculature. The tumors formed by the parental cell line, both in CAM and in mice, presented a clear distinct morphology from the ones formed by organotropic cells, demonstrating a more Matrigel-enriched component, where organotropic cell lines formed more compact tumors. Indeed, the presence of a microenvironment is the major advantage of in vivo models, such as the CAM and mice, that contrasts with cancer cell-derived models such as tumor organoids. The expression analysis of the breast CSC markers CD44 and CD49f by immunohistochemistry (IHC) confirmed the in vivo recapitulation of the stem cell features. CAM tumors present stronger staining in organotropic cell lines in comparison with the parental 231. Thus, the CAM system provides the formation of breast cancer-derived tumors that reproduce more faster and accurately the characteristics of CSC-enriched cell models used in this study when compared with mice model.

In a generic way, the in vivo CAM model shows several advantages compared to mice model, namely, easy handling, low cost, accessibility and rapid growth, reproducibility, reliability (reflected in a higher statistical power) and the absence of ethical and regulatory constraints. In particular, for the study of CSCs, we propose the LDA in the chick CAM model as an alternative, appealing and novel research tool to study CSC activity. Although our results are focused on breast cancer, we envisage that the CAM-LDA can be applied to other cancer cell models. However, this should be further validated, where different CSC markers should be selected according to the chosen cancer cell type. This system can still be further explored to test the efficacy of potential anti-CSC drugs with the potential to be used in a personalized medicine context.

## 4. Materials and Methods

### 4.1. Cell Culture

The breast cancer cell line MDA-MB-231 (231) was obtained from ATCC (American Type Culture Collection, Manassas, VA, USA), while its organotropic variants lung-metastatic (MDA-MB-231.LM2) [34], bone-metastatic (MDA-MB-231.BoM) [33] and brain-metastatic [MDA-MB-231.BRMS [32]) clones were obtained from J Massagué, the MSK Cancer Center, New York City, NY, USA. The four cell lines were maintained in Dulbecco’s minimal essential media (DMEM), supplemented with 10% fetal bovine serum (FBS), I and with 1% antibiotic solution—penicillin/streptomycin (all reagents from Invitrogen, Carlsbad, CA, USA). All the cell lines were cultured at 37 °C in a humidified atmosphere with 5% CO_2_ and used in experiments upon reaching 70–80% confluence.

### 4.2. In Silico Bioinformatics Analysis

To perform the in-silico analysis, the previously described gene expression profiles for significant deregulated genes (DEGs) in brain, bone and lung metastatic breast cancer cells were used [33,34,35]. After, these gene lists were crossed in order to obtain specific or shared DEGs for each metastatic breast cancer cell line. From this analysis, 80.8% (194 out of 240) of the genes were specifically deregulated in brain metastatic breast cancer cells (BCC), compared with 70.5% (60 out of 85) and 62.1% (59 out of 95) specifically deregulated genes for bone and lung metastatic BCC, respectively. Gene ontology (GO) terms were retrieved from http://amigo.geneontology.org/amigo/search/bioentity?q=stem.

### 4.3. Cell Surface Marker Analysis by Flow Cytometry

The expression of CD44 and CD24 on the cell surface of breast cancer cells was determined by flow cytometry analysis. Flow cytometry analysis for these markers was performed as previously described [3].

### 4.4. Presto Blue Assay

Presto blue assay was performed as previously described [3]. A minimum of 3 independent biological experiments were performed.

### 4.5. Mammosphere Assay

Mammosphere-forming assay was performed as previously described [3]. A minimum of 5 independent biological experiments were performed.

### 4.6. Limiting Dilution Assay in the In Vivo CAM Model

The chicken embryo CAM model was used to evaluate the tumorigenic response. Fertilized chick (*Gallus gallus*) eggs were obtained from commercial sources and incubated horizontally at 37 °C in a humidified atmosphere and referred to the embryonic development day (EDD). According to the European Directive 2010/63/EU, ethical approval is not required for experiments using embryonic chickens. Correspondingly, the Portuguese law on animal welfare does not restrict the use of chicken eggs for experimental purposes.

In detail, on EDD3, a square window was opened in the shell after the removal of 1.5–2 mL of albumen to allow detachment of the developing CAM. The window was sealed with a transparent adhesive tape and the eggs returned to the incubator. On EDD9, different numbers of MDA-MB-231 cells and their organotropic variants (resuspended in 5 uL of Matrigel and 5 uL of serum-free medium) were engrafted on top of the growing CAMs. Cells (1 × 10^6^ (1-M), 1 × 10^5^ (100-K), 1 × 10^4^ (10-K) and 1 × 10^3^ (1-K)) were inoculated, per embryo, into a 5-mm silicone ring under sterile conditions. The eggs were resealed and returned to the incubator for an additional 7 days, until EDD16. At this time point, the ring was removed; the CAM was excised from the embryos and photographed ex ovo under a stereoscope at 20× magnification (Olympus, Tokyo, Japan, SZX16 coupled with a DP71 camera). The area of CAM tumors was determined using the Olympus cell Sens Standard 1.14 program. Excised CAMs were fixed in 10% neutral-buffered formalin, paraffin-embedded for slide sections and stained with hematoxylin–eosin for histological examination or processed for immunohistochemistry.

### 4.7. Limiting Dilution Assay in the In Vivo Mice Model

For the Limiting dilution assay, a subcutaneous injection of the different cancer cells (231, 231.LM2, 231.BoM and 231.BRMS) in the flanks of female N:NIH(S)II-nu/nu mice, 6–8 weeks of age, was performed, using a 25-G needle. In detail, 3 to 4 mice per group and per condition were injected with consecutive cell dilutions ranging from 1 × 10^6^–1 × 10^4^ cells resuspended in Matrigel (1:1). Mice follow-up and tumor volumes were followed, as previously described [3], for a total of 3 weeks. At the end of the experiment, tumors were surgically removed, fixed in 10% buffered formalin, embedded in paraffin, sectioned and stained with hematoxylin and eosin.

The mice were housed, bred and maintained at the i3S Animal House in a pathogen-free environment under controlled conditions of light and humidity. All the experiments were conducted with the application of the 3Rs (replacement, reduction and refinement) (JP_2016_02 Project, animal ethics committee and animal welfare body of i3S).

### 4.8. Extreme Limiting Dilution Analysis (ELDA)

ELDA software was used to assess the number of self-renewing cells contained within each breast cancer cell line in both the in vivo CAM and mice models. ELDA has the capacity to calculate frequencies for stem cell subpopulations that produce 0% or 100% tumor engraftment, and, therefore, it is preferred for calculating tumor initiation from limited cell numbers. The ELDA software application analyses provided 95% confidence intervals and *t*-test statistical values that distinguished between the numbers of tumor initiating cells from the distinct conditions tested—http://bioinf.wehi.edu.au/software/elda/ [35].

### 4.9. Immunohistochemistry

Immunohistochemistry for CD44 and CD49f was performed in 3-μm sections of both excised CAM tumors and in mice xenografted tumor sections, as previously described [3].

### 4.10. Statistical Analysis

GraphPad version 5.0c software (GraphPad Software, San Diego, CA, USA) was used to construct the graphs and to perform the statistical analysis. An ANOVA test was used to calculate the significance of the mammosphere-forming efficiency (followed by Tukey’s test) and tumor growth in the CAM and mice assays (followed by Dunnett’s test). Flow cytometry experiments were carried out three times, with data pooled from all 3 experiments. Values of *p* < 0.05 were considered to be statistically significant.

## Figures and Tables

**Table 1:** Stem cell frequency in chorioallantoic membrane (CAM) tumors. Limiting dilution data as the percentage of tumor-forming eggs. The frequency of cancer stem cells (CSCs) was estimated using the Extreme Limiting Dilution Analysis (ELDA) calculating website. Confidence choice entered was 0.95, and *p*-values reflect differences in CSC frequencies between the metastatic cell lines (231.LM2, 231.BoM and 231.BRMS) and the parental cell line (231). N per group varied between 8–18 eggs for a total of 191 eggs inoculated.

Cell Model	Cell Number				Stem Cell Frequency (ELDA)	*p*-Value (Vs 231)
	1 M	100 K	10 K	1 K		
231	18/18 (100%)	5/9 (55.6%)	2/10 (20%)	3/8 (37.5%)	1/68,769	-
231.LM2	18/18 (100%)	10/11 (90.9%)	6/10 (60%)	4/10 (40%)	1/17,209	0.00163
231.BoM	18/18 (100%)	11/11 (100%)	7/11 (63.6%)	3/9 (33.3%)	1/7490	<0.0001
231.BRMS	18/18 (100%)	10/10 (100%)	4/10 (40%)	3/10 (30%)	1/12,215	0.00067

**Table 2:** Stem cell frequency in mice tumors. Limiting dilution data as the percentage of tumor-forming mice. The frequency of CSC was estimated using the ELDA calculating website. Confidence choice entered was 0.95, and *p*-values reflect differences in CSC frequencies between each of the organotropic cell lines (231.LM2, 231.BoM and 231.BRMS) and the parental cell line (231). N per group varied between 2–4 mice per group for a total of 39 subcutaneously injected mice.

Cell Model	Cell Number			Stem Cell Frequency (ELDA)	*p*-Value (Vs 231)
	1 M	100 K	10 K		
231	2/2 (100%)	2/4 (50%)	0/3 (0%)	1/15,7144	-
231.LM2	3/3 (100%)	4/4 (100%)	0/3 (0%)	1/37,558	ns
231.BoM	3/3 (100%)	4/4 (100%)	2/3 (66%)	1/9099	0.0041
231.BRMS	3/3 (100%)	4/4 (100%)	2/3 (66%)	1/9099	0.0041

## Data Availability

Not applicable.

## Supplementary Materials

The following are available online at https://www.mdpi.com/1422-0067/22/1/334/s1: Figure S1: CAM and mice xenograft images at lower magnification. Table S1: Significantly deregulated genes for each organotropic-derived breast cancer cell line.

## Abbreviations

231	parental MDA-MB-231 breast cancer cells
231.BRMS	brain-metastatic MDA-MB-231
231.LM2	lung-metastatic MDA-MB-231
231.BoM	bone-metastatic MDA-MB-231
APC	allophycocyanin
BCC	breast cancer cell
CAM	chorioallantoic membrane
CSCs	cancer stem-like cells
DEGs	deregulated genes
EDD	embryonic development day
ECM	extracellular matrix
ELDA	extreme limiting dilution assay
FBS	fetal bovine serum
GO	gene ontology
H&E	hematoxilin–eosin
HRP	horseradish peroxidase
IHC	immunohistochemistry
LDA	limiting dilution assay
MFE	mammosphere-forming efficiency
TICs	tumor-initiating cells
