# Supplementary material for: The Chick Chorioallantoic Membrane Model: A New In Vivo Tool to Evaluate Breast Cancer Stem Cell Activity
Source: Int J Mol Sci. 2020 Dec 30;22(1):334. doi: 10.3390/ijms22010334 (PMC7795925; doi:10.3390/ijms22010334)

Supplementary data

The Chick Chorioallantoic membrane model: a new *in vivo* tool to evaluate cancer stem cell activity

Supplementary table 1. Significantly deregulated genes for each organotropic derived breast cancer cell lines

ORGAN	LUNG		BONE		BRAIN					
GENE LIST	**94 genes (Ref 34)**		**85 genes (Ref 33)**		**240 genes (Ref 32)**					
DEREGULATED GENES (DEG)	ANGPTL4	CNOT2	AOX1	SIX2	ADAM19	KHDRBS3	WDR8	ENTPD3	PPP2R1B	PTGS2
	ARHGDIB	COL1A1	APM2	SLC4A7	ADAM8	KLHL25	WT1	ETS2	PRSS2	RARRES3
	ASMTL	COL6A1	ARHGEF10	SLC5A3	ADCY9	KTELC1	ZFP36L2	FAM20B	PRSS3	ROBO1
	CASK	CRSP2	BCL3	SLIM1	AGR2	LAMA4	ZMAT5	FAM26B	PSG7	SERPINE2
	CASP1	CSF2RA	CAV2	SOS1	AK5	LAMP3	ZNF140	FAM59A	PTPRU	SPINK4
	CLDN4	CXCL1	ChGn	SPG4	AKR1C1	LEF1	ZNF76	FCHO1	RAPGEF1	TNFSF10
	CSF1	DCAMKL1	CX3CL1	ST3GALVI	AKR1C2	LIF	ADAMTS1	FGFR1OP	RGS2	MMP1
	CSF3	EFEMP1	CYP1B1	STATI2	ALDH1A3	LOC100131795	CLU	FKBP5	RNF113A	IL32
	CST7	EMP1	DDR1	TGFBI	ALOX5AP	LOC100133916	COL5A1	FOXA2	RUNX1	JAG2
	DAAM1	EPHX1	DDX-10	TMEM4	APPL2	LOC100134294	CRADD	FPR1	S100A4	KCNK1
	EDIL3	EREG	DLC1	TNFAIP2	ARHGEF5	LOC100134295	CTGF	FZD1	SAT1	KCTD12
	FSCN1	GPR153	ENDOGL1	TNKS	ARPC1A	LOH11CR2A	CTSB	GABRE	SDC2	TTC15
	IL13RA2	GSN	EST	TRB	ASB1	LPHN2	DUSP1	GAL3ST4	SEPP1	TUBGCP3
	ISG20	GSTM4	FHL1	TUBA1	ASRGL1	LPXN	FGF5	GALC	SERPINB2	TXNIP
	JAG1	GYPC	FKBP11	ADAMTS1	ATP2B4	LRRC61	GBP2	GARNL4	SERPIND1	VEGFA
	KIAA1199	HDHD1A	FN1	CLU	B4GALT6	LSS	IL11	GBP1	SERPINE1	
	KYNU	ID1	FST	COL5A1	BAZ1B	MAGEC1	ITGB4	GJA1	SERPINI1	
	LTBP1	IDH2	FTH1	CRADD	BIN1	MAGEH1	NAP1L3	GJB3	SH3GL1	
	LY6E	IGSF4	FYN	CTGF	BMP4	MANSC1	NEDD4L	GMFG	SLC25A14	
	MAN1A1	KLRC1	GALNAC4S-6ST	CTSB	BRF2	MAP2K3	POMZP3	GOLSYN	SMAD1	
	OLFML2A	KLRC2	GPRC5C	DUSP1	C13orf15	MAPRE2	PON2	GSTP1	SNTB1	
	PDGFA	KRTHB1	H2AFL	FGF5	C17orf91	MED14	S100A2	H2BFS	SOX4 SRY	
	PTGS2	LAPTM5	H2AFO	GBP2	CASP4	MEF2C	SCNN1A	HBEGF	SPDEF	
	RARRES3	LBH	H2BFJ	IL11	CAV1	MMP3	ANGPTL4	HCFC1R1	SRGN	
	ROBO1	LOC221810	HFE	ITGB4	CCL20	MOCS1	ARHGDIB	HIST1H1C	ST3GAL1	
	SERPINE2	MAGED4	HLA-DRA	NAP1L3	CD99	MORC4	ASMTL	HIST1H1D	ST6GALNAC5	
	SPINK4	MATN2	IL15	NEDD4L	CDT1	NCK2	CASK	HIST1H2AC	STC1	
	TNFSF10	MBNL2	KHDRBS3,	POMZP3	CEBPD	NDUFB2	CASP1	HIST1H2BC	STS	
	MMP1	MBTPS2	KRT7	PON2	CENTD1	NINJ2	CLDN4	HIST1H2BD	STYXL1	
	ABCC3	MFAP2	LAMB1	S100A2	COL13A1	NMU	CSF1	HIST1H4A	SULT1B1	
	CXCR4	MMP2	MCAM	SCNN1A	COL18A1	NR3C2	CSF3	HIST2H2AA3	SULT1C2	
	HLA-DPA1	MOCOS	MITF	ABCC3	COPZ2	NUP160	CST7	HIST2H2BE	SUMO3	
	HLA-DPB1	MYH10	MRPS18B	CXCR4	CPVL	ODZ3	DAAM1	HLA-C	SUSD5	
	HLA-DRB3	NEDD9	N33	HLA-DPA1	CRIP2	PBX1	EDIL3	HLA-F	SYNJ2BP	
	SOX4	NGFB	NCF2	HLA-DPB1	CST1	PCBP3	FSCN1	HLA-G	SYNPO	
	SPANXB1	NNMT	PCTK2,	HLA-DRB3	CTDSPL	PCDH7	IL13RA2	HLA-J	TAGLN	
	SPARC	NR2F1	PFN2	SOX4	CTSC	PCTK1	ISG20	HOXC4	TCF3	
	ALDH3A1	PARD6B	PPL	SPANXB1	CUGBP2	PELI1	JAG1	HSPA12A	THBS3	
	APOBEC3G	PTPRN2	PRG1	MMP1	DAB2	PELI2	KIAA1199	HSPB1	TLR4	
	ARNT2	QPCT	PTK7		DLEU2	PFKL	KYNU	IDS	TMEM156	
	ATF1	ROR1	RHBDL2		DTNA	PHC1	LTBP1	IGFBP1	TNFRSF10D	
	ATP11A	SLC22A1LS	S100A3		EDN1	PHGDH	LY6E	IL18	TRIM24	
	C10ORF116	SLCO4A1	SAA2		EDNRA	PITX1	MAN1A1	IL1A	TRIM34	
	C14orf139	SPANXC	SCAMP4		ELAVL4	PLOD2	OLFML2A	IL1B	TRRAP	
	C4BPB	STOM	SE57-1		ENPP4	POLR3G	PDGFA	IL27RA	TSPAN1	
	C6orf108	TBC1D4	SERPINA1							
	ZNF185	TNC								

Supplementary Figure 1. CAM and mice xenographs images at lower magnification (10x)

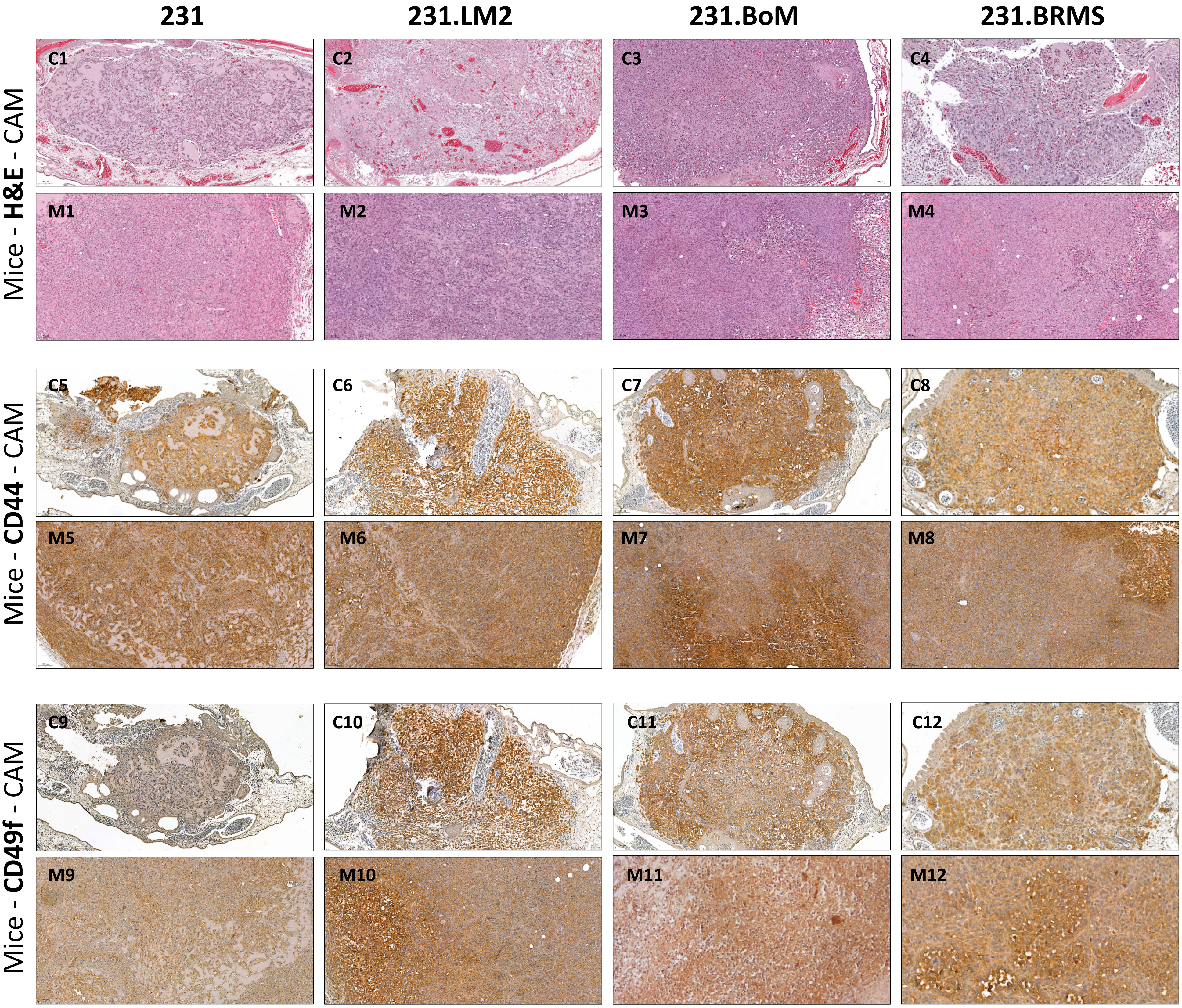

Supplement: Supplementary file 1 [file ijms-22-00334-s001.pdf]
